# Immediate Effect of Self-Modelling with Internal Versus External Focus of Attention on Teaching/Learning Gymnastics Motor-Skills

**DOI:** 10.2478/hukin-2022-0103

**Published:** 2022-11-08

**Authors:** Asma Amri-Dardari, Bessem Mkaouer, Samiha Amara, Sarra Hammoudi-Nassib, Hamdi Habacha, Fatma Zohra BenSalah

**Affiliations:** 1 Department of Individual Sports, Higher Institute of Sport and Physical Education of Kear Said, Manouba University, Manouba Tunisia; 2 Physical Education and Sport Sciences Department, College of Education. Sultan Qaboos University. Muscat, Sultanate of Oman; 3 Tunisian Research Laboratory “Sport Performance Optimization” of the National Centre of Medicine and Science in Sport, Tunis, Tunisia; 4 Université de Paris, CNRS, Integrative Neuroscience and Cognition Center, F-75006 Paris, France; 5 Faculty of Medicine of Tunis, University El Manar, Tunis, Tunisia

**Keywords:** parallel bars, video-modeling, feed-back, attentional focus

## Abstract

The aim of this study was to identify the immediate effect of self-modelling with different focus of attention strategies (i.e., internal vs. external) on the teaching/learning of gymnastics motor-skills. Fifty-nine non-gymnast students participated in this study and were divided into three groups (i.e., an external focus group (EF), an internal focus group (IF), and a control group (CG)). Each participant’s performance of the back dismount in the parallel bars was assessed before the experiment (i.e., base-score), and each participant was asked to provide a self-evaluation of their performance and their efficiency percentage. Afterwards, participants received a specific learning session (i.e., self-modelling with external focus, self-modelling with internal focus, or traditional learning with verbal instruction) and performed the back dismount in the parallel bars again immediately after (i.e., final score). Four international judges evaluated performance of our participants. The results showed that the EF and IF outperformed the CG in the final score. Importantly, a significant difference between the base and the final score was observed in the EF and IF, but not in the CG. In addition, the EF showed the highest percentage of improvement (Δ-score) and self-estimation scores compared to the two other groups. In conclusion, this study supports the adoption of external focus of attention for teaching/learning gymnastics motor-skills.

## Introduction

There are different means that can enhance performance of athletes practicing gymnastics and can equally lead them to reflect on their learning, communicate better and have a more professional production (Potdevin et al., 2018). As feedback on performance is also a vital factor, we can think of several instruments that can drive that feedback forward; for example, a computer and a webcam, a simple tablet, a smartphone, as well as the coach’s/teacher’s verbal instructions. All these instruments can serve as forms of immediate feedback in order to enhance athletes’ attention and consequently improve their performance (Eberline and Richards, 2013). For example, Porter et al. (2010) suggested that teachers could have a significant impact on performance by a simple modification of few words in the verbal instruction they give to students.

Several studies have confirmed that the focus of attention induced by the instructions or feedback provided to learners can have a significant impact on motor-skill learning (McNevin et al., 2003; Porter et al., 2019; Tsetseli et al., 2016a, 2016b). Teachers/coaches can use two-types of attentional focus strategies with their learners/athletes. The first is called an internal focus of attention and consists of providing instructions that direct learners/athletes to think about their own specific movements or body parts while executing a motor action (Marchant et al., 2007). The second strategy is referred to as an external focus of attention and consists of thinking about the effects of movements on the surrounding environment rather than own body parts (Wulf, 2007). According to Wulf et al. (2001), focus on the own body during execution of movement (i.e., internal focus of attention) could disrupt learning and motor performance, while focus on the environmental effects of movements (i.e., external focus of attention) could help improve motor skills (Wulf, 2007; Wulf et al., 2000, 2001, 2002).

In accordance with these findings, Benjaminse et al. (2018) suggested that instructions for visual and/or verbal external focus of attention enhanced transfer to another task, which is a very powerful method for motor learning. Shafizadeh et al. (2013) showed that external focus of attention facilitated learning of motor-skills even in situations where learning was by observation. In artistic gymnastics, Abdollahipour et al. (2015) revealed that performance of basic skills could be improved relatively easily by appropriate external focus instructions. Van Abswoude et al. (2018) suggested that the immediate effect of both types of focus of attention (i.e., internal and external) could directly enhance motor performance.

In addition, Wulf, Chiviacowsky et al. (2010) showed that the observational practice (i.e., video-modelling) provided athletes with information about the goal of movement and potential mistakes to avoid. That is, self-modeling via feedback on previous movement executions seemed to influence the efficacy of focus of attention strategies. Thus, combining video feedback and verbal instructions with internal and/or external attentional focus could have a considerable effect on learning.

Therefore, the purpose of the present study was to investigate the immediate effect of self-modelling with internal vs. external focus of attention on teaching/learning a basic gymnastics skill in non-gymnast students. We chose the back dismount in the parallel bars as a learning element. We hypothesized that self-modelling combined with the external focus of attention could lead to better performance than with the internal focus of attention.

## Methods

### Participants

Fifty-nine male non-gymnast physical education and sport science students divided into three groups (i.e., an external focus group (EF): age 21.26 ± 1.29 years, body height 1.78 ± 0.02 m, body mass 69.37 ± 5.86 kg; an internal focus group (IF): age 20.26 ± 2.03 years, body height 1.76 ± 0.06 m, body-mass 72.05 ± 3.88 kg; a control group (CG): age 21.52 ± 0.78 years, body height 1.77 ± 0.04 m, body mass 71.27 ± 4.92 kg) and one expert high-level gymnast (age 22 years, body height 1.67 m, body mass 58 kg) agreed to participate in this study. The three groups (i.e., three classes of first-year students in the sciences and techniques of physical activities and sports) of the same level followed the same training program (i.e., the official program of the higher institute of sport and physical education) under the same working conditions.

Participants were in good health, without muscular, neurological or tendon injury. After being informed in advance of the procedures, methods, benefits, and possible risks of the study, each participant had to review and sign a consent form to participate in the study. The experimental protocol was performed in accordance with the Declaration of Helsinki for human experimentation and was approved by the local Ethics Committee of the National Centre of Medicine and Science in Sport (LR09SEP01).

### Experimental Design and Procedures

**Figure 1:**
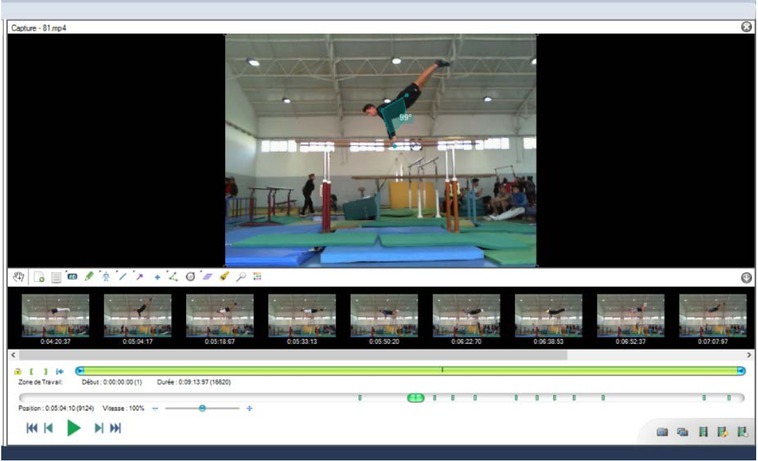
Self-modelling with Kinovea® software.

This study was planned over two sessions per class, (i.e., six sessions), to observe the immediate effect of different focus of attention strategies (i.e., internal vs. external) on the teaching/learning gymnastics skills improvement in the parallel bars (i.e., back dismount). During the first session, four international judges evaluated performance of each student/group when performing a back dismount in the parallel bars over a regulator height (i.e., 1.80 m) prescribed by the FIG (Figure 1). This note was considered as a base score (BS). During the second session (e.g., spaced by 24 hours and at the same hour of the day), a visualization of the own gesture (e.g., self-modelling) was established using the freeware Kinovea 8.15 [www.kinovea.org/en/downloads/] (Puig-Diví et al., 2019) for each student/group (Figure 1) accompanied or not (i.e., the CG) by a concentration strategy (i.e., internal or external focus of attention) following groups (i.e., IF and EF). This note was considered a final score (FS). From this score we calculated the percentage of real efficiency (RE).

*External Focus Group (EF)*: After viewing their performances, followed by corrective instructions, students in this group were invited to direct their attention to the effect of their movements on the environment. Instructions were to focus on external landmarks (e.g., parallel bars, the ceiling of the room) in order to correct/perform the movement (e.g., swing up above the bars, pay attention to each end of the bars, increase the height of the swing toward the ceiling, rise vertically during the swing, raise the feet above the bars, look at the carpet landmark for a good landing).

*Internal Focus Group (IF)*: Following their performances and provided with corrective instructions, students in this group were required to direct their attention to their own movements. Instructions were to focus on their bodies/segments (e.g., opening of the arm-trunk angle, body sheathing, segmental placement) to correct/perform the movement (e.g., keep arms and legs straight, sheathe the body, open the body to the maximum, do not bend the arms, open the angle between the arm and the trunk, align the shoulder, the trunk, and feet).

*Control Group (CG)*: After watching their own performances, followed by corrective instructions, students in this group were asked to correct/perform the movement following standard verbal instructions (e.g., go up as strong as possible, the highest possible, as fast as possible; swing harmoniously; raise the feet as high as possible; dismount with flexibility).

### Self-estimation and evaluation

An estimated score (ES) was obtained from participants after they judged their own achievement of the back dismount on the parallel bars (i.e., self-assessment) via an evaluation score of 0 to 10 points. In addition, each participant judged the quality of his progress, and an estimated efficiency percentage (EE) was determined for his performance on the parallel bars (i.e., from 0 to 100%).

### Assessment model

**Figure 2:**
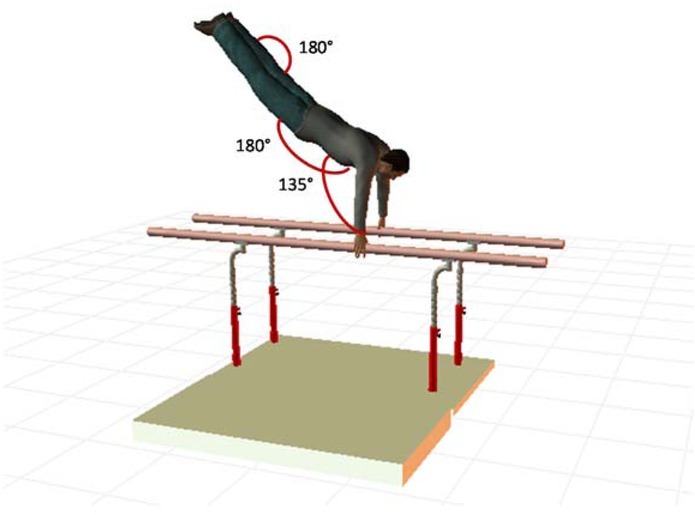
Reference position when performing the back dismount on the parallel bars.

The evaluation model was established from the kinematic analysis of a high-level expert gymnast, assisted by four international men’s artistic gymnastics judges and based on the FIG code of points shown in Table 1 and Figure 2.

**Table 1:** Parallel Bar Scoring System.

	Arms/Trunk ang (°) 4 pt.	Trunk/Leg ang (°) 3 pt.	Thigh/Leg ang (°) 3 pt.
Excellent (Full score)	Shoulder angle ≥ 135	Pelvis angle = 180	Knee angle = 180
Small Error (-0.5 pt.)	120 ≥ Arms/Trunk ang < 135	Trunk/Leg ang ≥ 165 < 180 and/or Trunk/Leg ang ≤ 195 < 180 (180 ± 15) *	Thigh/Leg ang ≥ 165 < 180
Medium Error (-1 pt.)	115 ≥ Arms/Trunk ang < 120	Trunk/Leg ang ≥ 150 < 165 and/or Trunk/Leg ang ≤ 210 < 195 (180 ± 30) *	Thigh/Leg ang ≥ 135 < 165
Large Error (-2 pt.)	90 ≥ Arms/Trunk ang < 115	Trunk/Leg ang ≥ 13 < 150 and/or Trunk/Leg ang ≤ 225 < 210 (180 ± 45) *	Thigh/Leg ang ≥ 90 < 135
Fall # (Non- recognition)	Arms/Trunk ang ≤ 75	Trunk/Leg ang ≥ 120 < 135 and/or Trunk/Leg ang ≤ 240 < 225 (180 ± 60) *	Thigh/Leg ang ≤ 75

(*) In flexion or hyper extension; (#) On the bars or during the landing

### Statistical Analysis

Statistical analysis was conducted via SPSS 20.0 software [SPSS. Chicago. IL. USA]. Descriptive statistics (means ± SD) were performed for all variables. The normality of distribution estimated by the Shapiro-Wilk test was not acceptable for all variables. Consequently, nonparametric tests were used for comparative statistics. The Kruskal-Wallis H test and the Mann-Whitney U test were applied for between groups comparison, and Friedman's ANOVA followed by the Wilcoxon's rank-sum test for within groups comparison. Additionally, effect sizes for nonparametric tests (r) were calculated with the formula *r* = Z / √N. The following scale was used for the interpretation of the size effect: *r* > 0.10 [small]; *r* > 0.30 [medium]; *r* > 0.50 [large] (Berben et al., 2012). Similarly, the delta variation (Δ-score) between the base score and the final score "Δ (%) = [(FS - BS) / BS] × 100" was calculated in order to estimate the percentage of variation between the two scores (DS). A priori level less than or equal to 0.5% (*p* ≤ 0.05) was used as a criterion for significance.

## Results

The Kruskal-Wallis H test revealed a significant main effect of group (i.e., EF, IF and CG) on the final score (χ^2^= 17.393, *p* < 0.001, *r* = 2.174), on the real efficiency (χ^2^= 13.999, *p* < 0.001, *r* = 1.749), and on the delta score (χ^2^= 14.028, *p* < 0.001, *r* = 1.753). Accordingly, the EF and IF improved their final score (3.86 ± 1.06 pts vs. 5.43 ± 1.39 pts with ICC = 9.20, CV = 0.248 and 3.22 ± 1.03 pts vs. 5.00 ± 0.76 pts with ICC = 0.896, CV = 0.237, respectively) to a greater extent than the CG (3.12 ± 1.23 pts vs. 3.84 ± 1.13 pts with ICC = 0.924, CV = 0.196). Pairwise comparisons using the Mann-Whitney U test are presented in Table 2.

**Table 2:** Mann-Whitney U Test, between groups pairwise comparison score.

		Mann-Whitney U	Z	Sig.	*r*
Final Score (FS)	EF vs. IF	141.000	-1.427	0.154	0.226
	EF vs. CG	103.500	-3.128	0.002**	0.495
	IF vs. CG	99.500	-3.684	0.000**	0.582
Real Efficiency (RE)	EF vs. IF	147.500	-1.238	0.216	0.196
	EF vs. CG	115.000	-2.822	0.005**	0.446
	IF vs. CG	116.000	-3.302	0.001**	0.522
Delta Score (DS)	EF vs. IF	118.000	-2.031	0.042*	0.321
	EF vs. CG	87.000	-3.326	0.001**	0.526
	IF vs. CG	144.000	-2.486	0.013*	0.393

(EF) External focus group; (IF) Internal focus group; (CG) Control group; (*) Significant at p < 0.05; (**) Significant at p < 0.01; r > 0.1 [small]; r > 0.30 [medium]; r > 0.50 [large].

For the kinematic analyses of the back dismount (i.e., arms/trunk, trunk/legs, and thigh/leg angles), a Kruskal-Wallis H test was run on the base score and the final score with the attention focus group as a between-subjects factor (i.e., EF, IF and CG). The main effect of group was significant for the arms/trunk angle in both the BS (χ^2^= 8.320, *p* < 0.05, *r* = 1.040) and the FS (χ^2^= 7.768, *p* < 0.05, *r* = 0.971). The same in the thigh/leg angle in both the BS (χ^2^= 6.633, *p* < 0.05, *r* = 0.829) and the FS (χ^2^= 8.522, *p* < 0.05, *r* = 1.065). Accordingly, the EF and IF improved their technical performance (arms/trunk angle 81.24 ± 15.17° vs. 92.52 ± 13.58° with ICC = 0.952, CV = 0.048 and 77.16 ± 12.55° vs. 80.60 ± 13.32° with ICC = 0.721, CV = 0.080; thigh/leg angle 133.71 ± 18.09° vs. 147.76 ± 22.30° with ICC = 0.751, CV = 0.068 and 136.72 ± 20.81° vs. 139.40 ± 17.61° with ICC = 0.637, CV = 0.088, respectively) to a greater extent than the CG (arms/trunk angle 77.44 ± 14.07° vs. 78.72 ± 17.81° with ICC = 0.509, CV = 0.119 and thigh/leg angle 127.44 ± 17.50° vs. 131 ± 19.63° with ICC = 0.514, CV = 0.084). Pairwise comparisons of angular kinematic data using the Mann-Whitney U test are presented in Table 3.

**Table 3:** Mann-Whitney U Test, kinematic analysis between groups pairwise comparison.

		Mann-Whitney U	Z	Sig.	*r*
Arms/Trunk angle BS	EF vs. IF	206.000	-.468	0.640	0.075
	EF vs. CG	114.000	-2.115	0.034*	0.323
	IF vs. CG	138.500	-2.739	0.006**	0.404
Thigh/Leg angle BS	EF vs. IF	147.500	-1.910	0.056	0.306
	EF vs. CG	98.500	-2.551	0.011*	0.389
	IF vs. CG	240.000	-.497	0.620	0.073
Arms/Trunk angle FS	EF vs. IF	221.000	-.099	0.921	0.016
	EF vs. CG	113.000	-2.143	0.032*	0.327
	IF vs. CG	144.000	-2.615	0.009**	0.386
Thigh/Leg angle FS	EF vs. IF	171.500	-1.318	0.188	0.211
	EF vs. CG	93.000	-2.706	0.007**	0.413
	IF vs. CG	172.000	-1.996	0.046*	0.294

(EF) External focus group; (IF) Internal focus group; (CG) Control group; (BS) Base score; (FS) Final score; (*) Significant at p < 0.05; (**) Significant at p < 0.01; r > 0.1 [small]; r > 0.30 [medium]; r > 0.50 [large].

Friedman's ANOVA revealed a very significant difference between the different scores (i.e., BS, FS and ES) for the EF (χ^2^= 26.955, *p* < 0.001, *r* = 6.353), the IF (χ^2^= 24.222, *p* < 0.001, *r* = 5.286), and the CG (χ^2^= 34.400, *p* < 0.001, *r* = 6880). Pairwise comparisons between the different scores and efficiency (i.e., EE and RE) using the Wilcoxon's rank-sum test are presented in Table 4.

**Table 4:** Wilcoxon Signed Ranks Test, scores pairwise comparison.

Groups		Estimated Score vs. Base Score	Final Score vs. Base Score	Estimated Score vs. Final Score	Real Efficiency vs. Estimated Efficiency
External Focus Group	Z	-3.651	-3.866	-2.429	-1.930
(EF)	Sig.	0.000**	0.000**	0.015*	0.056
	*r*	-0.861	-0.911	-0.573	0.455
Internal Focus Group (IF)	Z	-3.597	-4.110	-1.644	-1.871
	Sig.	0.000**	0.000**	0.100	0.061
	*r*	-0.785	-0.897	-0.359	0.408
Control Group (CG)	Z	-4.257	-1.626	-4.126	-3.920
	Sig.	0.000**	0.084	0.000**	0.000**
	*r*	-0.851	-0.325	-0.825	0.784

(*) Significant at p < 0.05; (**) Significant at p < 0.001; r > 0.1 [small]; r > 0.30 [medium]; r > 0.50 [large].

The pairwise comparison (i.e., Wilcoxon's rank-sum test) of the angular kinematic variables in the BS and the FS showed that the arms/trunk angle was significantly improved in the EF and IF (*p* < 0.01; *r* = 0.553). In addition, the trunk/leg angle was significantly improved only in the EF (*p* < 0.05; *r* = 0.337). On the other hand, the arm/trunk angle showed no significant improvement for the three groups.

## Discussion

The aim of the present study was to investigate the immediate effect of self-modelling with the internal vs. the external focus of attention on the teaching/learning of a basic gymnastics motor-skill. Three groups of participants were recruited, and they performed a back dismount movement on the parallel bars before receiving a training session to record their base score (BS). The first group (EF) followed a training program including self-modeling combined with the external focus of attention instructions, the second group (IF) completed a training program including self-modeling combined with the internal focus of attention instructions, while the third group (CG) underwent a traditional training program with standard verbal instructions. Immediately after, the three groups performed the same movement, and their final scores were recorded.

According to Shafizadeh et al. (2013) and Porter et al. (2010), coaches can have a significant impact on performance by simply changing one or two words of the verbal instructions given to their athletes. Our findings confirmed that conclusion by revealing an improvement in the final execution scores in participants using different strategies of attentional focus (i.e., EF and IF), but not in participants who did not use a strategy of the focus of attention (CG). Furthermore, participants using the external focus of attention showed a higher performance improvement than the two other groups. This result is in agreement with numerous studies that observed the beneficial effect of an external relative to an internal focus of attention on motor skills learning (Gorgovan, 2021; Hijazi, 2013; Lawrence et al., 2011; Singh and Wulf, 2021). Moreover, our results showed that, while there was no difference in final scores between the external and internal focus groups, scores in the internal focus of attention group were higher than in the control group. This finding suggests that, even if the external focus of attention seems to be the best strategy to improve performance, the internal focus of attention still guarantees better performance than with no instructions.

Accordingly, our results confirm the beneficial effect of attentional focus on learning motor skills and further corroborate the advantage of external attentional focus (i.e., focus oriented towards the objective or the consequences of movements) over internal attentional focus (i.e., focus oriented towards the movement itself) (Land et al., 2014; Makaruk et al., 2020; Wulf, 2007; Wulf, Shea, et al., 2010). This could be explained by a higher degree of automaticity and less conscious interference linked to external focus of attention compared to internal focus (Wulf et al., 2001).

Moreover, the advantages of external focus of attention seem to be often immediate, during the acquisition period (Porter et al., 2010; Wulf and Dufek, 2009; Wulf and Su, 2007). In accordance with this suggestion, our results show an immediate improvement in the quality and precision of the back dismount execution: in the alignment of the body during the backswing with the opening of the arms/trunk and pelvis angles, as well as at the level of the landing on the ground with precision. These results are consistent with the results of An et al. (2013), Wulf, Chiviacowsky, et al. (2010), and Wulf et al. (2007), suggesting that external attentional focus improves reliability and precision of movement execution.

On the other hand, the comparison of the real vs. estimated efficiency percentages showed a significant difference only in the control group. The other groups (i.e., EF and IF) were able to fairly estimate their efficiency percentages. This may be due to the fact that focus of attention could help participants to direct their attention to the most relevant aspects of the movement in order to have more hindsight and critical reflecting on the movement and enhance its execution with respect to own body parts and to its relation with the surrounding environment. An athlete who watches her/his own movements and evaluates his/her motor skills, combined with appropriate instructions from the coach, could have more information to rely on in order to correct the execution and improve it (i.e., self-modelling). The coaches’ task here is no longer filming athletes in order to judge their performance, but rather to encourage them to auto-evaluate their skills and to make a reflective feedback. This type of reflection seems to be an effective self-assessment tool in the learning process (Rolheiser et al., 2000).

Several studies on motor learning have demonstrated the importance of self-modeling as a very effective teaching tool. It allows both the teacher/coach and the learner/athlete to improve their knowledge of the body in motion and its mental representation during the didactic act (Le Naour et al., 2019). In addition to corroborating the effect of self-modeling on learning, our study suggests that combining it with instructions of focus of attention could make it more efficient. That is, after judging the execution and the consequences of a movement, its amplitude, and its rhythm, both by coaches and athletes, instructions for an external or internal attentional focus could be better oriented and thus improve motor learning.

We should acknowledge several limitations in the present study related to the motion analysis system used. Future studies could use a full kinematic analysis with a real-time motion analysis system with muscle modeling, as Sinclair et al. (2015). In addition, it would be interesting to adopt a multidisciplinary approach that brings together motor control, movement analysis and teaching/learning gymnastics skills as suggested by Farana et al. (2021).

## Conclusion

Overall, the results of the present study support the adoption of an external attentional focus strategy for the execution and improvement of a motor-skill such as the back dismount on parallel bars. Our results suggest that the use of self-modelling combined with verbal instructions to direct participants’ attention outward significantly improves motor-skill performance. Thus, we suggest that coaches/teachers could direct their instruction and feedback to external aspects in the training/learning process and provide athletes/learners with additional relevant information derived from video-feedback and modeling.
